# Disparities in cervical cancer screening programs in Cameroon: a scoping review of facilitators and barriers to implementation and uptake of screening

**DOI:** 10.1186/s12939-023-01942-2

**Published:** 2023-08-17

**Authors:** Namanou Ines Emma Woks, Musi Merveille Anwi, Taal Bernard Kefiye, Dohbit Julius Sama, Angel Phuti

**Affiliations:** 1 Institute of Tropical Medicine and International Health, Charité Universitätsmedizin, Berlin, Germany; 2 Faculty of Medicine and Biomedical Sciences, University of Yaoundé I, Yaoundé, Cameroon

**Keywords:** Cervical cancer, Screening, Inequity, Cameroon, Barriers, Facilitators

## Abstract

**Background:**

Cervical cancer is the fourth most common cancer worldwide. Organized screening has achieved significant reductions in cervical cancer incidence and mortality in many high-income countries (HICs). But the gap between HICs and low-and-middle-income countries (LMICs) is still substantial as the highest burden of the disease is in LMICs. Cameroon is a LMIC, where cervical cancer is the leading cause of cancer-related deaths among women, only 3–5% of eligible women have been screened and there is no effective national cervical cancer prevention program.

**Objective(s):**

Identify facilitators and barriers to the implementation and uptake of existing cervical cancer screening programs in Cameroon to inform the implementation of a comprehensive national program.

**Methods:**

We conducted a scoping review using the Preferred Reporting Items for Systematic Reviews and Meta-analysis, extension for Scoping Reviews (PRISMA-ScR). Google Scholar and five electronic databases (PubMed, CINAHL, Embase, Cochrane library and Web of Science) were searched systematically from 2012 to 2022. Articles on cervical cancer screening programs in Cameroon were eligible for inclusion. Two reviewers independently screened search results and extracted relevant data.

**Results:**

A total of 182 articles were identified using our search strategy, and 20 were included. There was scarcity of publications from the North, Adamawa, East and South regions of Cameroon. Barriers and facilitators found were presented using the World Health Organisation framework for health systems. Cross-cutting barriers were: (1) the lack of a national training curriculum for screening providers with no elaborate, harmonized screening and treatment algorithm for cervical precancers; and (2) women’s lack of information about cervical cancer screening activities. Conversely, provision of screening services at a low or no cost to women in some programs and the feasibility of using novel point of care screening methods like the Human Papillomavirus DNA test were identified as facilitators.

**Conclusion:**

This scoping review indicates that there are knowledge and research gaps concerning the state of cervical cancer screening services in some regions of Cameroon. Moreover, it underlines the need for comprehensive cancer control policies and practices integrating all six-health system building blocks to reduce disparities between regions, and rural versus urban areas in Cameroon.

**Supplementary Information:**

The online version contains supplementary material available at 10.1186/s12939-023-01942-2.

## Introduction/Background

Cervical cancer is preventable, yet it is the fourth most common cancer and a leading cause of mortality among women worldwide [1]. In 2020, over 600,000 new cases of cervical cancer were identified, representing 6.5% of all newly diagnosed cancers in women globally [1]. According to the International Agency for Research on Cancer (IARC), the highest burden of this disease is in sub-Sahara Africa, especially in low-and-middle-income countries (LMICs) [2]. Meanwhile, high-income countries (HICs) have achieved significant reductions in incidence and mortality rates through the implementation of organized and financially accessible screening programs [3–5]. Consequently, there is a substantial gap in mortality rates due to cervical cancers between HICs and LMICs, as about 90% of the estimated 342 000 cervical cancer-related deaths in the world, in 2020 occurred in LMICs [6].

Cameroon is a Sub-Saharan African and a LMIC, with cervical cancer as the leading cause of female cancer deaths, and an estimated 1,787 deaths in 2020 [2]. Cameroon’s healthcare system is divided into three sectors: the public, private and traditional sectors. Health institutions in the public and private sectors are responsible for organising cervical cancer screening programs. Some programs either conduct screening routinely or sporadically, meaning during special days or events [7, 8]. The Cameroon Baptist Convention Health Services (CBCHS) is an institution in the private health sector which runs the Women’s Health Program (WHP) which is currently the only coordinated, multicentre, routine cervical cancer prevention program in Cameroon [9]. There are presently ten CBCHS facilities found in six of ten regions in Cameroon with a functional WHP [10].

To accelerate the elimination of cervical cancer worldwide, the World Health Organisation calls on all countries to implement a three-pillar approach to reduce incidence rates to less than 4 per 100 000 women by 2030 [11]. This approach entails fully vaccinating at least 90% of girls by age 15, screening at least 70% of women by age 35 and again by age 45, and treating at least 90% of women diagnosed with precancer and invasive cancer [11]. To date, only 3 – 5% of eligible women have been screened for cervical cancer in Cameroon and there is currently no effective national cervical cancer prevention program [12]. As a result, there is inequitable access to cervical cancer screening across the territory, with women living in areas outside the capital cities having lower odds of screening for cervical cancer [12].

Hence, there is an urgent need for an effective strategy to increase awareness and perform timely screening and treatment of premalignant cervical lesions in women nationwide. An overview of the country’s health system barriers and enablers timely, nationwide screening of cervical precancerous lesions in women have to be known to succeed. Therefore, a preliminary search of published literature was made to identify studies on cervical cancer screening in Cameroon, but neither a scoping review nor a comprehensive assessment of cervical cancer screening programs in the nation was found.

### Research question(s)

1. What are the barriers and facilitators to existing cervical cancer screening programs in Cameroon?
2. What are the recommendations to researchers and the Ministry of Public health for the design of a comprehensive national cervical cancer screening program?

### Objective(s)

1. Summarise existing research evidence on cervical cancer screening in Cameroon.
2. Identify barriers and facilitators to existing cervical cancer screening programs in Cameroon in order to inform a national cervical cancer screening program.
3. Make recommendations to researchers and the ministry of public health for the design of a national cervical cancer screening program.

## Methods

### Study type, protocol registration and reporting guidelines

We conducted a scoping review to systematically map available literature on the topic, synthesise findings, and identify key concepts and gaps [13]. Our research protocol was drafted using the Joanna Briggs Institute (JBI) protocol template for scoping reviews, revised by the research team and registered with the Open Science Framework (OSF) to ensure transparency and reduce research duplication. The OSF provides a collaborative management platform for researchers to conduct, share, and report their research [14]. This review has been reported using the Preferred Reporting Items for Systematic Reviews and Meta-analysis, extension for Scoping Reviews (PRISMA-ScR) [13].

### Eligibility criteria

**Inclusion criteria:** Articles written in English or French language, published between 2012 and 2022 and conducted in Cameroon on cervical cancer prevention.

**Exclusion criteria**: Duplicates and papers without focus on cervical cancer screening or treatment of precancerous lesions, articles centred on diagnostic accuracy, reports or reviews.

### Procedure

The scoping review was conducted by two independent reviewers and a final peer reviewer in five main stages.

### Literature search

The customised search strategy for electronic databases was drafted based on the keywords: cervical cancer prevention, cancer screening, cryotherapy, Cameroon, conization, cervical intraepithelial neoplasia (CIN), visual inspection with acetic acid (VIA) and human papillomavirus (HPV). This strategy was drafted by the first reviewer and later revised by the final peer reviewer using the Peer Review of Electronic Search Strategies (PRESS) checklist [15]. The PRESS 2015 checklist has six items: translation of the research question, Boolean and proximity operators, subject headings, text word searching, “spelling, syntax and line numbers”, limits and filters [15].

The three-step approach below was used to build the electronic database search strategy for PubMed:

- A preliminary search was conducted in PubMed using its controlled vocabulary called Medical Subject Headings (MeSH) and the keywords (in English and French): cervical cancer prevention, cervical cancer screening, cryotherapy, conization, cervical intraepithelial neoplasia, CIN, VIA, HPV and Cameroon to find synonyms and other spellings of the keywords.
- Boolean operators such as OR and AND were incorporated, truncation (using the asterisk * to find variations of words with multiple endings), nesting (using brackets to group similar terms separated by the Boolean operator OR), quotation marks (to demarcate phrases) and field tags like [tw], [tiab] and [All fields] were applied progressively to combine keywords and MeSH terms.
- The search strategy was run in PubMed to determine its validity and detect errors.

The search strategy used in PubMed is presented below:

1. “Uterine cervical neoplasms”[MeSH] OR “Cervical intraepithelial neoplasia”[MeSH] OR “Uterine cervical dysplasia”[MeSH] OR “cancer du col de l’utérus”[All fields] OR “cervical carcinoma*”[All fields]
2. “Early detection of cancer”[MeSH] OR “cryotherapy”[MeSH] OR “conization”[MeSH] OR “Papanicolaou Test”[MeSH] OR “colposcopy”[MeSH] OR “Human Papillomavirus DNA Test*”[MeSH] OR “screen-and-treat”[All fields] OR prevent*[All fields] OR dépistage[All fields] OR “thermal ablation”[All fields] OR LEEP[All fields] OR LLETZ[All fields] OR “Cold Knife”[All fields]
3. “Cameroon”[MeSH] OR “Cameroun”[All fields]
4. #1 AND #2 AND #3

It was translated to four other databases [Cumulated Index to Nursing and Allied Health Literature (CINAHL), Excerpta Medica Database (EMBASE), the Cochrane library and Web of science] and Google scholar to conduct similar searches. In Google scholar, the first 100 results obtained were screened for eligibility. See Additional file 1 for the search strategies used in other databases.

#### Removal of duplicate publications

The final search results were imported into the Endnote 20 software, where duplicates were removed by the first reviewer. Publications retained after the removal of duplicates were exported to Microsoft Excel 365 software and shared with the second reviewer.

#### Title and abstract screening

The first and second reviewers screened the titles and abstracts of retained papers independently using the screening sheet (See Additional file 2) in Microsoft Excel 365 to exclude off-topic articles. Disagreements on study inclusion or exclusion were all resolved by discussion. References with only titles or only abstracts were moved to the next stage for retrieval of full texts.

#### Full-text screening

Full texts of articles included were then retrieved and placed in a group called “full text screening” in EndNote 20. The Endnote file containing all retrieved articles was shared with the second reviewer. Full texts were assessed for eligibility in EndNote independently by the first and second reviewers. All reviews or reports were excluded at this stage, because the research team believed that they would be more useful in the discussion of findings. At this stage, disagreements were resolved through discussions between the first and second reviewers. Reviewers discussed until a consensus of 80% or more was attained concerning the list of articles to include in the review. Articles retained after the consensus were included in the study. Finally, the reference/citation lists of all included articles were searched to find other relevant publications to add to the scoping review.

#### Data extraction

Data was extracted from studies using the data charting forms in Additional file 3 using Microsoft Excel 365. The first and second reviewers charted the data independently, discussed the results, and came to a consensus about what needed to be reported in the results section. Any disagreements between the two reviewers were resolved through discussion or further mediation by the final peer reviewer. The form was designed to capture specific details on the characteristics of each source of evidence (article), the screening and treatment methods used during screening programs and factors influencing the implementation of cervical cancer screening programs.

## Results

### Literature search and characteristics of included studies

**Fig. 1:**
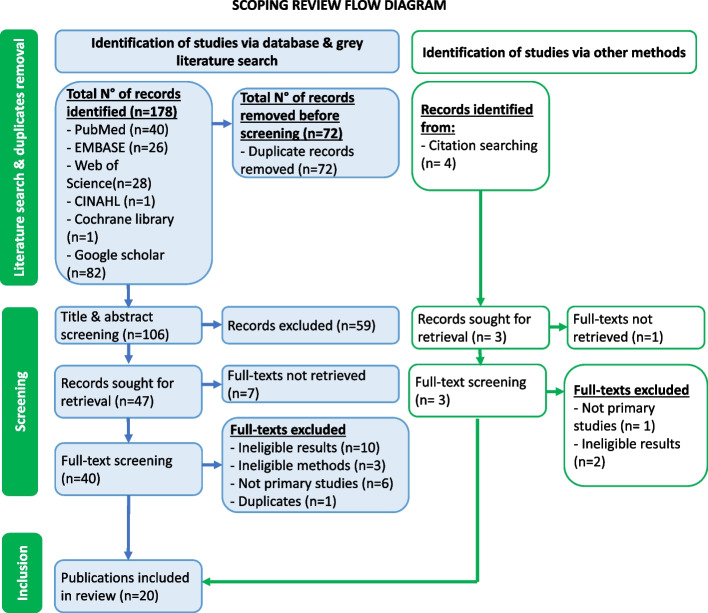
Scoping review procedure illustrated with the PRISMA 2020 flow diagram

A total of 182 publications were identified using our search strategy; 178 in five databases and grey literature (Google Scholar) and four in the reference lists of included papers. One hundred and six scientific papers proceeded to title and abstract screening following the removal of duplicates. At this stage, 59 publications were excluded, while 47 were retained. Amongst the exclusions, three had neither titles nor abstracts, one was a study protocol, 10 focused on diseases other than cervical cancer and 45 were centred around concepts different from cervical cancer screening programs (diagnostic accuracy, vaccination, disease prevalence and knowledge about cervical cancers). Hereafter, full texts were successfully retrieved for 40 of 47 papers and screened. Then, 20 articles were excluded for reasons provided in Fig. 1. Fourteen studies reported in 20 publications fulfilled eligibility criteria and were included [9, 16–34].

Each article included in the review at this stage had its reference list screened for other eligible publications. Four scientific papers qualified for full-text retrieval. Full-text screening found none of the four eligible for inclusion. The arguments for this decision are stated in Fig. 1. Therefore, in total, 20 publications from 14 studies were included in the scoping review. See Table 1 for the characteristics of included studies.

**Table 1:** Characteristics of included studies (per administrative region)

Study	Administrative region (location)	Type of study	Type of participants	N° of participants	Methods of data collection & screening	Previous screening exposure	Screening uptake	Post-screening treatment	Follow-up
**B. Wabo et al. 2019** [30]	Centre region (YUTH, YGOPH)	Cross-sectional study	Women (25–65 years)	523 women	Questionnaires No screening	209/523 (40%)	N/A	N/A	N/A
**J.S. Dohbit et al. 2018** [31]	Far North region (seven hospitals with maternity unit in Maroua)	Cross-sectional study	Post-partum women (25–45 years)	622 women	Questionnaires No screening	16/622 (2.6%)	N/A	N/A	N/A
**J.F. Domgue et al. 2020** [25]	North-west region (Seven remote villages)	Community intervention	Non-pregnant women (30–65 years)	1292 women	Case forms Screening (self-HPV, VIA/VILI-DC in HPV -positive)	60/1292 (4.7%)	1270/1292 (98.3%)	Thermal ablation (161/182 = 88.5%) LEEP (1/1)	- Women treated with thermal ablation to follow-up > 1 year
**L. Donatus et al. 2019** [16]	North-west region (Kumbo-west HD)	Cross-sectional study	Women 25–65 years	253 women	Questionnaires No screening	110/253 (43.48%)	N/A	N/A	N/A
**G.E. Halle-Ekane et al. 2018** [29]	South-west region (University of Buea)	Cross-sectional study	Male & female students > 16 years	416 students (208 males, 208 females)	Questionnaires No screening	10/208 (4.8% did Pap smear)	N/A	N/A	N/A
**A. Adedimeji et al. 2021** [23]	South-west region (Limbe Regional Hospital)	Qualitative study	Women (≥ 25 years) and men	80 women 20 men	08 FGDs 08 IDI Screening (HPV-self collection)	None (due to inclusion criteria)	N/A	N/A	N/A
**A. Tassang et al. 2020** [21]	South-west (Buea Regional Hospital Annex)	Cross-sectional study (Intervention)	Women ≥ 21 years	140 women	Questionnaire Screening (VIA/VILI-colposcopy)	37/124 (29.8%)	124/140 (88.6%)	Thermocoagulation (0/1 treated) LEEP (1/1 treated)	Follow-up for negative cases after 03 years
**C. Eakin et al. 2018** [19]	South-west region (University of Buea)	Cross-sectional study (Intervention)	Women ≥ 21 years (faculty, staff and students)	120 women	Questionnaires Screening (VIA/VILI)	39/120 (32.5%)	115/120 (95.8%)	Thermocoagulation (5/5) LEEP (2/2 received)	- Low-grade lesions in 21–24 years to repeat screening > 1 year
**P.M. Tebeu et al. 2020** [24]	West region (health facilities in Mifi HD)	Cross-sectional study	HCPs (MD, nurses, midwives)	200 HCPs	Questionnaires No screening	N/A	N/A	N/A	N/A
**M. Pham et al. 2022** [33] **& P. Hämmerli et al. 2022** [32] **& J. Levy et al. 2020** [28] **& A.M. Datchoua et al. 2021** [22] **& A.N. Roux et al. 2021** [17]	West region (Dschang HD)	Community intervention	Women 30–49 years	1940 women in Dschang or surroundings	Questionnaires Screening (selfHPV, VIA/VILI in HPV +)	N/A	100%	- Thermal ablation - LLETZ/LEEP	N/A
		Qualitative study	CHW aged 21–77 years	11 CHWs	11 IDI	N/A	N/A	N/A	N/A
		Community intervention	Women 30–49 years	840 women	Case report forms Screening (self-HPV, VIA/VILI in HPV + women	N/A	100%	- Thermal ablation (100%) - LEEP	After 5 years if HPV- After 1 year if HPV + & VIA/VILI - After 1 & 6 months if treated
		Qualitative study	Women 30–49 years and men	31 women 12 men	6 FGD Screening (self-HPV)	N/A	N/A	N/A	N/A
	West region (Dschang & Mbouda HD)	Qualitative study	HCP working in Dschang & Mbouda	16 HCPs	04 FGD Questionnaires	N/A	N/A	N/A	N/A
**M. Viviano et al.** [26] **& M. Kunckler et al. 2017** [20]	West region (Dschang HD)	Community intervention	Women 30–49 years	1012 women	Data collection forms Screening (self-HPV, VIA/VILI)	N/A	HPV-100% VIA/VILI in 98.9% of HPV +	- Thermal coagulation	Repeat screening > 5 years if HPV neg –Control > 1 month if treated
**R.T. Simo et al. 2021** [18]	West region (Djeleng & King Palace centres)	Intervention	Asymptomatic women (23–60 years)	228 women	Questionnaires Screening (Pap smear)	4/228 (1.75%)	182/228 (79.8%)	N/A	N/A
**P. Cholli et al. 2017** [34]	South-west & Littoral(Mutengene & Mboppi Baptist hospitals)	Prospective (Intervention)	Women ≥ 30 years	1170 women	Questionnaires Screening(clinician HPV & VIA/VILI-DC co-testing)	N/A	100%	- Thermal ablation, LEEP - Cryotherapy Total: 18/44	Participants’ phone numbers collected
**G.DeGregorio et al. 2016 & 2017** [9, 27]	07 CBCHS WHP hospitals (multiregional)	Retrospective study (2007–2014)	HIV + women ≥ 21 years HIV-negative if ≥ 25 years	44 979 women	Medical history Screening (VIA/VILI-DC)	N/A	44 979/46 048 (97.7%)	- Same-day cryotherapy – 219/705(31.1%) - LEEP-unknown	VIA/VILI inadequate advised to return after one year for follow-up

*YUTH* Yaoundé University Teaching Hospital, *YGOPH* Yaoundé Gynaeco-Obstetric and Paediatric Hospital, *N/A* Non-applicable, *HPV* Human Papillomavirus, VIA*/VILI* Visual Inspection with Acetic Acid/Visual Inspection with Lugol’s Iodine, *DC* Digital cervicography, *LEEP* Loop Electrosurgical Excision Procedure, *ICC* Invasive Cervical Cancer, *HD* Health district(s), *FGD* Focus Group Discussion, *IDI* In-depth interview, *MD* Medical doctor, *HCP* Healthcare provider

*N/A* Non-applicable, *LLETZ* Large Loop Excision of the Transformation Zone, *LEEP* Loop Electrosurgical Excision Procedure, *CHW* Community Health Worker, *HD* Health district, *HPV* Human Papillomavirus, VIA*/VILI* Visual inspection with acetic acid/ Visual inspection with lugol’s iodine, *FGD* Focus Group Discussion, *IDI* In-depth interview, *HCP* Healthcare provider, *CBCHS* Cameroon Baptist Convention Health Services, *WHP* Women’s Health Program, *HIV* Human Immunodeficiency Virus, *HPV* + HPV positive, *HPV*—HPV negative, VIA*-DC* Visual inspection with acetic acid-digital cervicography, *VILI-DC* Visual inspection with lugol’s iodine-digital cervicography

### Facilitators and barriers to the implementation of cervical cancer screening programs

**Fig. 2:**
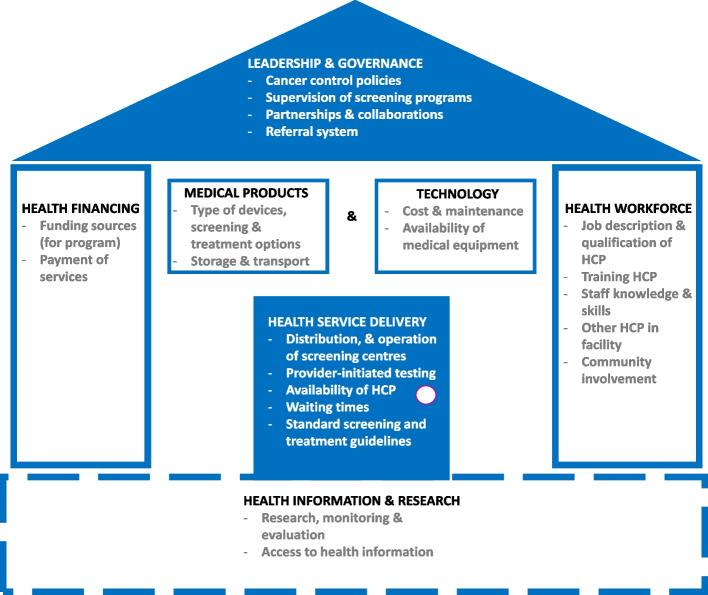
Determinants of successful implementation of cervical cancer screening in Cameroon with WHO’s health system blocks

The barriers and facilitators identified in this review have been presented and discussed using the framework of WHO’s health system building blocks (See Fig. 2).

### Leadership and governance

Leadership-related enablers to the successful implementation of cervical cancer programs identified by this review are described below.

#### Inadequacies related to cancer control policies

Women interviewed by Adedimeji et al*.* mentioned the lack of comprehensive policies that can reduce rural–urban disparities in availability of preventive healthcare services and encourage timely uptake of cervical cancer screening by women [23].

#### Absence of supervision for screening programs

In the WHP study, 33.3% (18/54) of women did not receive same-day treatment for premalignant lesions of the cervix because no supervisor was available to review their VIA-DC/VILI-DC images [9]. Of four articles which highlighted the role of supervision in cervical cancer screening programs, only the one cited above mentioned its impact on the success of screening programs [9]. On the flip side, CHWs’ accountability to a senior staff in the program could play an enabling role in their task of sensitizing women to screen for cervical cancer [32]. This is shown in the comment below from a female worker [32]:

“*Because…….there are some cases of refusal, but when we come to see him [a doctor in the screening program], he gives us more ideas to go and convince the person.*” (Maka04)

#### Partnerships or collaboration

Different public–private partnerships provided the foundation to expand cervical cancer screening programs. The partnership established between the universities of Arizona and Buea; and the CBCHS provided expertise for sporadic screen-and-treat programs for cervical precancerous lesions [19, 21]. Geneva university hospitals and the universities of Dschang and Yaoundé worked together to assess the suitability of innovative cervical cancer screening options like HPV DNA self-sampling for Cameroonian women in Dschang [26]. In this study, local staff were trained by physicians from Geneva and Dschang through an e-learning platform and practical sessions on-site to provide cervical cancer screening services [28].

Conversely, Adedimeji and collaborators [23] highlighted the extensive focus of public health resources on health programs like HIV/AIDS to be a major reason for insufficient attention to other important issues like cervical cancer. Moreover, many cancer screening programs in this review were initiated primarily by private or international institutions [23], some in partnership with public institutions, but with little or no information about the transition plan from international leadership to government ownership for sustainability [26, 32].

#### Poor referral system

Healthcare providers interviewed by Roux et al*.* in Dschang indicated that medical referral systems were inadequate [17]. An example of this challenge was mentioned in another study where a patient diagnosed with ICC could not receive complete treatment (radiotherapy and radical hysterectomy) in a single referral facility and needed to be managed in two different facilities [25]. Although the radical hysterectomy was performed by a trained gynaecologist, the patient died shortly after, probably due to postoperative complications [25].

### Health workforce

The health workforce-strategies identified as barriers and facilitators to the success of cervical cancer screening programs in this review are noted hereafter.

#### Community involvement

A cross-sectional, interventional study conducted in the Dschang health district observed a 30.1% (584/1940) increase in women’s attendance at the cervical cancer screening program following recruitment by community health workers (CHWs) [33]. These findings are similar to women’s participation in screening programs at 35.9% (1292/3600) in another study in where Community Mother and Child Aids (CoMCHAs) sensitized women about cervical cancer screening [25]. Farmers and housewives, who are sometimes difficult to reach were more represented in the CHW-led recruitment at 42.6% and 25.2% [33].

#### Job description & qualification of HCPs

Specific roles with clearly defined responsibilities played an important role in the success of WHP and Dschang cervical cancer screening programs [27]. CHWs in Dschang distributed invitation vouchers to each woman they approached, such that these vouchers were presented by women at their cervical cancer screening visits [33]. It enabled traceability to distinguish between the impact of CHWs and communication and information channels [33]. In the WHP, nurses screened and treated women for premalignant lesions of the cervix, performed biopsies and referred women in need of higher level of care to the appropriate facilities [9, 27]. LEEP was usually performed by LEEP-certified nurses in the WHP. Providers’ proficiency in native languages facilitated the implementation and uptake of cervical cancer screening, as trained field workers could explain the stepwise self-collection process of vaginal specimens for HPV DNA tests to women in the local dialect and pidgin English [25].

#### Training HCPs

Little information was provided about formal, pre-employment training required to be a cervical cancer screening staff in these studies. However, each program had a distinct training curriculum for front-line providers of cervical cancer screening services to facilitate program implementation. The training curriculum of the WHP was adapted from the Cervical Cancer Prevention Program in Zambia (CCPPZ) [19, 25, 27, 28, 33, 34].

#### Limited knowledge and skills

Deficiencies in levels of staff knowledge, technical and communication skills were reported in several studies, and these are potential barriers to the success of cervical cancer screening programs. A cross-sectional study assessing the knowledge and practices of HCPs in Mifi, West region found that only 35% of 200 participants knew that HPV infection was a risk factor for cervical cancer [24]. Concerning VIA/VILI, a substantial proportion (22.0%) of results were inadequate in the retrospective study of the WHP [9]. The ectocervix stained negatively in these cases, but the clinician was unable to visualise the entire squamocolumnar junction. In Mifi, West region, few healthcare workers were knowledgeable about common cervical cancer screening and treatment options; 26% knew about VIA, 39% had heard of VILI, 22.5% were aware of the existence of the HPV DNA test, 17% were knowledgeable about cryotherapy and 9% had knowledge on conisation; meanwhile, 80% knew about the Pap smear [24]. Amongst 11% of participants who mentioned that they had performed cervical cancer screening in women, a third (10/29) of medical doctors were counted, compared to a minority of (12/171) of paramedical professionals [24].

Furthermore, other potential workforce-related barriers to cervical cancer screening uptake found by Datchoua et al*.* were inadequate health communication skills and unethical treatment of women by HCP [22]. A female participant in this study shared:

*“You know others initially traumatise people. For example, the woman [referring to a female HCP] who was recording there, she […..] asks Poupoupou questions (brutally/quickly)! She stresses you out by asking the questions quickly. No, that’s not the way to do it.......”(Female P13B)*

#### Other HCPs in health facility

In a qualitative study exploring barriers to cervical cancer screening uptake, HCPs reported a lack of awareness and interest in the program among medical doctors practising in the Dschang district hospital [17].

### Health financing

#### Funding sources for screening programs

The financial resources needed to sustain cervical cancer screening programs were either provided by external grants or fees paid for services offered. The long-standing WHP relied essentially on a fee-for-service system, which seemingly facilitated sustainable implementation and to a limited extent on external donations [25, 27]. On the other hand, the 5-year long cancer screening program in Dschang was funded primarily by external grants from different institutions in Switzerland [33].

#### Payment for services

A recurrent observation in many studies was the non-negligeable proportion of women who had never been screened for cervical cancer because of poverty or high cost of services as a barrier to uptake. A cross-sectional study showed that about 6.32% (16/253) of women had never screened for cervical cancer because they found it quite expensive [16]. Poverty was reported as a barrier to cervical cancer screening uptake by 4.68% (8/173) and 14.3% (75/523) of women in studies conducted by Simo et al*.* and Wabo et al*.,* respectively [18, 30]. Moreover, a qualitative study of the cervical cancer screening program in Dschang showed that women were hesitant to turn up at the hospital for free cervical cancer screening due to transportation costs [17]. The following quote from a female hospital staff highlights this [17]:

*“It would have been impossible for me [and many of us] to show up for the free screening if not that we knew we would be given transport money for coming……..” (FGD, female 36–45 years)*

Furthermore, scarcity of publicly funded routine screen-and-treat programs for cancers of the cervix shifted women to private clinics, most of which offer these services at an exorbitant cost. One participant in a qualitative study reported [23]:

*“Private clinics are expensive and want to make as much money instead of providing appropriate care. I know of people who started going to a private clinic to receive care….” (FGD female, 36–45 years)*

With regards to facilitators, the retrospective study of the WHP shows that women who were unable to bear the cost of screening services were screened and treated in exchange for no fee, a reduced fee or payment later, based on their socio-economic status [9, 27].

### Service delivery

There are eight key characteristics of good service delivery in the guide for monitoring the WHO building blocks of health systems [35], but our study evaluated cervical cancer screening delivery using these five aspects below because the former is detailed and better obtained through primary studies.

#### Distribution and operation of screening centres

There was scarcity of data about the situation of cervical cancer programs in four (North, Adamawa, South and East regions) of ten regions in Cameroon. The main barrier highlighted by publications under this first subtheme was the low uptake of screening or treatment for cervical precancers among women living in far-to reach areas. Domgue et al*.* found that the treatment rate with thermal ablation was low (30.8%) in one of seven participating villages which was distant, in contrast with the overall rate (88.5%) [25].

Cervical cancer screening was offered by the WHP in a comprehensive package with other women’s health services, including family planning, sexually transmitted infections (STI) treatment, breast cancer screening, and vaccination [9, 25, 27]. Although this could be a potential enabler of successful implementation and uptake of cervical cancer screening, no study was found assessing the impact of this on the success of cervical cancer screening programs.

#### Standard screening and treatment guidelines

We found detailed program-specific guidelines for screening and treatment of cervical cancer and precancers, but no detailed national screening and treatment algorithm in Cameroon [19, 27].

#### Provider-initiated testing and counselling (PITC)

Tebeu et al*.* found that only about 15% of healthcare providers in the West region spontaneously proposed cervical cancer screening services to eligible women aged 35 years and above [24]. Findings by Simo et al*.* corroborate with this as only 7.89% of the study population heard about cervical cancer prevention from the medical staff, and Wabo et al*.* also found that 16.8% (88/523) of participants believed that not receiving a medical prescription for screening was a barrier to uptake [18].

#### Shortage of HCPs

The shortage of trained HCPs was highlighted by women as one of the most significant structural barriers during a cervical cancer screening program in the South-west region [23].

#### Long waiting times

The HCPs interviewed by A. N. Roux et al*.* recognized long waiting times to be a barrier to cervical cancer screening uptake [17]. An HCP from Dschang commented:

*“And some patients told us that it takes a lot of time. For them, it should be a 10 minute thing. But, they enter, they stay one hour at the informative causerie (informative causerie refers to the informative talk that is given to women to give information on cervical cancer prior to screening) ….and they wait for the results! (…). This prevents them from coming.” (Female staff)*

### Medical products & technology

#### Type of devices, screening & treatment options

CareHPV tests were used to screen women in the WHP due to their affordability over XpertHPV [25]. The latter delivered HPV type-specific results within an hour of analysing cervico-vaginal samples [26]. So, while using the screen-triage-and-treat approach on one-day consultations, this enabled women screened with cervical precancers to be treated by the healthcare team on the same day [20, 26, 28, 33]. Other contextual adaptations made to minimise costs and facilitate cervical cancer screening included the use of dry cervico-vaginal swabs [26], which required no refrigeration and self-collection of HPV samples [25]. In the study conducted by Domgue et al*.*, the availability of a generator made it possible to treat cervical precancerous lesions using thermocoagulation in the absence of electric current [25].

#### Storage & transport

The WHP screening program preferred thermocoagulation over cryotherapy devices to treat precancerous lesions of the cervix because of their portability [27].

#### Cost and maintenance

Carbon dioxide gas is needed for cryotherapy, and it was costly and hard to get [27]. Therefore, cryotherapy devices were sometimes defective when needed [27]. Equipment failure was the cause of deferred care in 8/54 (14.8%) patient records retrieved by the 8-year retrospective study done by the WHP [9, 27]. On the other hand, thermocoagulation did not require expensive and heavy carbon dioxide (CO_2_) tanks [27].

#### Non-availability of medical equipment

Women participating in the study conducted by Adedimeji et al*.* identified the limited supply of basic equipment for screening as a barrier to the successful implementation and uptake of cervical cancer screening [23]. Viviano et al*.* indicated that providers could not treat one patient due to impossibility of heating the thermocoagulation probe [26].

### Health information and research

#### Research, monitoring and evaluation

The use of electronic patient records over paper records in the WHP may have facilitated the 8-year long retrospective study of cervical cancer screening data [9, 27]. Nurses in the WHP met on a quarterly basis and reviewed snapshots of screened cervices with normal and abnormal findings in order to arrive at a consensus on strategies to improve the quality and interpretation of VIA/VILI-DC images of the cervix and the treatment and follow-up of women [27].

#### Access to health information

Poor access to relevant information about cervical cancer screening was a recurrent reason revealed by women for not screening for cervical cancers. Most cervical cancer prevention programs invited women to screen through the following channels: radio advertisements, talks and posters in health centres, churches, women’s associations, community gatherings, banners and social media campaigns [21, 28, 33, 34]. The enabling role of CICs in cervical cancer screening uptake is shown by studies conducted in the Centre and West regions of Cameroon which found 64.2% (336/523) and 13.6% (31/228) of screened women, respectively citing “the media” as a major source of information on cervical cancer screening [18, 30]. Phone calls were also used to notify women for results collection and a follow-up appointment [34]. A cross-sectional study exploring barriers towards Pap smear screening among university students in the South-west region found that 10.1% (21/208) of women lacked information on screening programs [29]. Other studies found 25.3% of women in the North-west, 68.9% in the West and 83.7% in the Far North regions who had never been screened for cervical cancer had also never been informed about cervical cancer screening [16, 18, 31]. Likewise, a majority of HCPs in the Dschang health district noticed a lack of awareness on cervical cancer among women, especially in rural areas where the levels of formal education were lower [17]. A female HCP in this study shared [17]:

*“And for many of them, even when you try to inform them, you realise how important the level of education is. They understand today, but they will forget tomorrow. Or maybe they will tell you that they understand and they don’t truly” (Female HCP)*

Participants of the screening program in Dschang also recognized that inadequate information about cervical cancer prevention, causes or symptoms was an important barrier [22]. Simo et al*.* further revealed that 3.1% of women who believed that it was not possible to prevent cervical cancer had received wrong information from family and friends [18]. Majority (60%) of women enrolled into a study at two hospitals in the Centre region had never been screened for cervical cancer, and 25.8% (135/523) of participants cited lack of information as a barrier to screening [30].

## Discussion

The primary purpose of this scoping review was to collate existing research evidence on cervical cancer screening programs in Cameroon. We identified 20 publications reporting factors impacting the implementation of cervical cancer screening programs in Cameroon in six of its 10 regions. In four (Adamawa, North, East, South) of these 10 regions, no eligible publications were found on cervical cancer screening programs, indicating that cervical cancer screening activities may be non-existent or rudimentary therein. These results differ from those of a comprehensive assessment of cervical cancer prevention in Zambia, which show that all 10 provinces have at least one hospital or district providing routine cervical cancer “screen-and-treat” services [36]. It is equally possible to make routine “screen-and-treat” services for cancers of the cervix available in all regions of Cameroon, since the country has previously had a coordinated scale-up of the HIV/AIDS “test-and-treat” services to all its 10 regions [37].

With regards to leadership and governance, a qualitative study included in this review mentioned the absence of policies aimed at promoting equity in service provision between urban and rural areas in Cameroon as a barrier to cervical cancer screening uptake [23]. Without a comprehensive evaluation of cervical cancer screening activities, the Ministry of Health will find it challenging to develop tailored policies to address existing gaps between rural and urban areas in practice [38]. Furthermore, approximately 33% (18/54) of women in the WHP eligible for same-day treatment did not receive it, because of the unavailability of a supervisor to review VIA-DC images and confirm treatment algorithms [9]. An evaluation of the cervical cancer screening program in 13 clinics in Indonesia had comparable findings by showing that regular availability of supervisors was essential to successful delivery of services [39]. Therefore, measures need to be put in place by screening programs to ensure that at least one supervisor is available when needed.

With regards to the health workforce, two Cameroon-based studies found a 30–36% increase in the number of women screened for cervical cancer following the involvement of CHWs [25, 33]. In Iran, the recruitment of women by CHWs raised cervical cancer screening rates from 0% to 62.85% over a period of two months [40]. This indicates that CHW contribute to increase cervical cancer screening uptake by women [32]. Furthermore, the WHP and cervical cancer screening project in Dschang had independent program-specific training curricula for frontline providers of screening and treatment of CIN [27, 28]. Unfortunately, with the above training approach in place, many HCPs (especially mid-level) in Cameroon were still found to have poor knowledge of visual and HPV-based cervical cancer screening options, with a substantial proportion of them unskilled in performing VIA/VILI [9, 24]. Unlike Cameroon, Britain has a national cervical cancer screening or colposcopy training and certification program provided by the British Society for Colposcopy and Cervical Pathology (BSCCP) [41]. This ensures that cervical precancer screening and treatment providers across the country develop harmonised and context-adapted communication, diagnostic and therapeutic skills. Frontline clinicians need to learn techniques which improve the visualisation of the SCJ of the cervix, especially in older women [42]. Co-testing with HPV or cytology, whenever VIA/VILI returns inadequate results could also address the problem of VIA/VILI-inadequate results and better inform treatment decisions. Women expressed concerns about poor communication and unethical behaviour of HCPs towards them during screening programs [22]. Lack of patient-centred communication was also identified as a barrier to women’s adherence to cervical cancer screening in Botswana [43]. Measures need to be in place to train and encourage HCPs to be more humane and patient with women in their practice.

The cervical cancer prevention program in Dschang was a research project, scheduled to run over a 5-year period and funded mainly by external donors [28, 33]. Grants for most research projects in Africa are obtained through partnerships with collaborators from Europe or North America, where the funding lies [44]. As a result, some routine cervical cancer programs existing in some African countries are completely funded by external donations, thereby raising questions about sustainability [45]. However, the WHP identified in this review is one of the exceptions whose major source of financing is client fees, with donors contributing quite little to the program’s budget [9]. The WHP’s positive experience of over 10 years suggests that the fee-for-service model can be sustainable if it provides a system for cost-recovery and is properly managed [27]. Two studies in this review found a similar proportion of women (4–6%) who had never been screened for cervical cancer before, because they considered screening services expensive [16, 18]. Furthermore, the elimination of user fees and the reimbursement of women’s transport fare was reported as a motivating factor for women to adhere to screening programs [17, 23]. This shows the need for contextual measures to be implemented to make women contribute to the user fees only to the extent that it will not lead them to financial hardship.

Concerning service delivery, it was clear from the studies included in this review that distance played a significant role as a disincentive for women to adhere to cervical cancer screening in Cameroon [17, 23, 25]. In a cervical cancer screening program, treatment rate was low (30.8%) in the village located over two hours away from the screening clinic was set up [25]. The losses to follow-up suggest the need to give women who live in far-to-reach areas the possibility to receive screening and treatment services for cervical precancers in a single clinic visit. Alternatively, as done in a community-based cervical cancer outreach in Kenya which registered a higher percentage (51%) of women with positive screens returning for treatment, phone calls or messaging could be used to inform and remind women about their results and follow-up appointments [46]. Moreover, countries like South Africa have set the standard national travel distance for primary health care (PHC) at 5 km [47]. There was no mention of the maximum distance separating currently existing clinics that offer screening and treatment services for precancers of the cervix in Cameroon. Our findings further show that clinicians rarely recommended cervical cancer screening to women [24, 30]. Meanwhile, Simms and colleagues illustrated that provider-initiated testing and counselling (PITC), which refers to the practice of routinely recommending HIV testing to people attending health facilities plays an important role in increasing the uptake of HIV testing [48, 49]. Proven benefits of this approach in the management of HIV/AIDS can be leveraged to improve women’s uptake of cervical cancer screening services. Women interviewed during a qualitative study in the South-west region mentioned that the shortage of HCPs limited their access to cervical cancer screening services [23]. Studies conducted in other African and European countries also identified staff shortages as a barrier to cervical cancer screening and highlighted the absence of female staff or trained staff during some periods as subfactors which hindered women from screening [50, 51]. In the same way, HCPs perceived that another factor which discouraged women, their peers and family members from turning up for screening programs was the long waiting time for results [17]. Therefore, regular surveys could be made to identify bottlenecks in administrative procedures and consider suggestions from staff and patients to reduce waiting times. Program-specific guidelines for cervical cancer screening provided clear details regarding recommended screening age group, follow-up and rescreening intervals, screening and treatment algorithms following cervical cancer screening [19, 25, 27]. Meanwhile, Cameroon’s national cancer control guidelines specify mainly the recommended age group to be screened for cervical cancers, without presenting a contextualised, evidence-based screening and treatment algorithm [38]. In contrast, Rwanda’s clinical guidelines for cervical cancer screening outline extensive information about practice recommendations [52]. In a systematic review comparing country-specific guidelines for cervical cancer screening, countries’ recommendations for cervical cancer screening practices were issued primarily by central decision-making bodies for the health sector, or in collaboration with national medical professional societies [53]. A similar pattern can be used by Cameroon to develop an elaborate, overarching, evidence-based, clinical guidelines for secondary prevention of cervical cancer.

As concerns medical products and technology, recent WHO recommendations suggest that cervical cancer screening programs should use HPV DNA tests as primary screening tests, with VIA/VILI, cytology, colposcopy or partial genotyping to triage women after a positive HPV DNA test [11]. Accordingly, some studies in this review screened women using either CareHPV or XpertHPV assays [25, 26]. Although CareHPV was low cost, it required at least two visits per screening episode, since it is designed to operate in batch mode, with optimal use at 90 samples per batch [25, 54]. This number of samples per day is achievable only by a few clinics in LMICs. On the flip side, XpertHPV is expensive, but it facilitated same-day treatment of cervical precancers in the cancer research project in Dschang, because it runs in non-batch mode and delivers results within an hour of analysis [54]. This could address potential loss to follow-up challenges associated with multiple hospital visits per screening episode. Hence, measures are required to obtain inputs from country experts in evidence-based medicine and disease prevention to inform national procurement decisions for quality assured, accurate and cost-effective HPV DNA assays. VIA-DC/VILI-DC returned high rates of inadequate results during the WHP, and the primary reason identified was limited skill of frontline staff and the absence of senior staff during a specific period [9]. The use of a smartphone to capture cervical images instead of a simple camera could allow for real-time support and hands-on training from senior screening staff who are not there in person to improve the accuracy of the test [55]. Studies in this review also showed that cryotherapy devices need CO_2_ supply from tanks which are difficult to transport, hence thermal coagulators were preferred over cryotherapy because of their portability [27]. A meta-analysis of studies conducted in LMICs showed that both cryotherapy and thermal coagulation are effective therapeutic modalities for CIN lesions [56]. Therefore, thermocoagulation is a suitable alternative to cryotherapy in this context. A limited supply of basic equipment was also identified in this review as a barrier to the success of cervical cancer screening programs [23]. This corroborates with reports about occasional non-availability of functional cryotherapy devices or CO_2_ gas supply in the WHP [27]. In the same way, a study conducted in Swaziland reported different types of screening equipment shortage [50]. Equipment shortages can lead to suspension of cervical cancer screening activities. Thus, the management of health facilities and screening programs need to set up effective systems for timely stockkeeping, procurement, and repair of medical equipment.

The capacity of the WHP’s health information system (HIS) to foster research and evaluation of care is seen in the program’s successful retrieval of data collected over an 8-year period, with clear communication of challenges and opportunities encountered [9, 27]. On the other hand, routine health information systems (RHIS) in Cameroon have been reported to be poor in developing research in health, promoting informed decision-making and disseminating health information [57]. Lessons can therefore be learned from the successes of the HIS used by the WHP to address some problems in the RHIS of other cervical cancer screening programs or health facilities in Cameroon.

Several CICs were used by cervical cancer screening programs to raise awareness among women and educate them about cervical cancer prevention [28, 33, 34]. Studies in this review showed that some women in the West and Centre regions had access to health information about cervical cancer screening through the media [18, 30]. This is probably due to the significant presence and operation of cervical cancer screening programs and awareness campaigns in these regions. However, there was still a substantial proportion of women in other regions of Cameroon who had never been screened for cervical cancer because they lacked adequate information [16, 18, 31]. Roux et al*.* found that lack of knowledge was more prevalent among rural dwellers, where the levels of formal education were lower [17]. In support of this result, the Far North region, which has the lowest education enrolment rate amongst other regions in Cameroon, had the highest percentage (83.7%) of women citing “lack of information” as a barrier to cervical cancer screening [31, 58]. Improving women’s literacy, especially in rural areas, could be a key step towards increasing their adherence to cervical cancer screening programs. This might require special strategies for different population groups based on their level of education and employment status. Some countries use specific websites to disseminate context-adapted, health messages to the general population, such as the Center for Disease Control (CDC) in the United States and the National Health Service (NHS) in England. Other supplementary strategies like the involvement of CHWs, peer educators, delivery of health education material in local dialects and mobile health technologies have proven their success in LMICs [33, 59]. Lessons can be learned from these examples to improve the dissemination of health information on cervical cancer prevention in Cameroon. Further exploration confirms that the absence of reliable sources of information about risk factors for cervical cancer, prevention strategies, the importance of timely screening, disease manifestations and prognosis, the geographical locations and working hours of screening centres in Cameroon act as an important barrier [17, 23, 60]. Therefore, health education is more beneficial when structured, from a reliable source to address the knowledge gaps of the population and clarify their myths and misconceptions about a subject.

## Study limitations and conclusion

There are a few limitations to our scoping review. First, we did not perform a risk of bias assessment for individual studies because it is a scoping review and not a systematic review. Further, our aim was to map out existing evidence to provide guidance to researchers and health system actors planning the implementation and expansion of cervical cancer screening programs in Cameroon. Second, some publications included in our scoping review were qualitative studies, therefore, the results may not be generalizable. However, other quantitative studies in this review and other countries had results which were consistent with findings of the qualitative studies.

A total of 182 articles on cervical cancer screening in Cameroon were identified by this scoping review. Of these, 20 publications from 14 studies/research projects were included. This review found barriers and facilitators to the implementation of cervical cancer screening programs in Cameroon and classified them using the WHO framework for health systems. Cross-cutting barriers identified were: (1) the lack of appropriate cancer control policies to reduce disparities between the country’s administrative regions, and rural versus urban areas; and (2) women’s lack of information about cervical cancer screening activities. Conversely, the provision of screening services at a low or no cost to women and the feasibility of using novel, speedy and possible point of care screening methods like HPV DNA test in Cameroon stood out in several publications as facilitators. The findings in this paper indicate that there are knowledge and research gaps concerning the state of cervical cancer screening services in the North, Adamawa, East and South regions of Cameroon. Moreover, there is no national training curriculum for frontline providers of cervical cancer screening, no elaborate and harmonized, national screening and treatment algorithm for cervical precancers in Cameroon.

### Supplementary Information

**Additional file 1.**

**Additional file 2.**

**Additional file 3.**

## Data Availability

The Excel spreadsheets generated for data synthesis during the current study are available from the corresponding authors on request.
