# Supplementary material for: Disparities in cervical cancer screening programs in Cameroon: a scoping review of facilitators and barriers to implementation and uptake of screening
Source: Int J Equity Health. 2023 Aug 17;22:156. doi: 10.1186/s12939-023-01942-2 (PMC10433640; doi:10.1186/s12939-023-01942-2)
Supplement: Supplementary file 1 — Additional file 1. [file 12939_2023_1942_MOESM1_ESM.docx]

**APPENDIX 1: Customised search strategies used in other sources of literature**

**EMBASE search strategy**

1. exp uterine cervix cancer/ di, pc [Diagnosis, Prevention]
2. exp uterine cervix dysplasia/ co, di, dm, ep, su, th [Complication, Diagnosis, Disease Management, Epidemiology, Surgery, Therapy]
3. cancer du col de l’utérus.mp.
4. cervical intraepithelial neoplasia.mp.
5. #1 OR #2 OR #3 OR #4
6. exp cancer screening/
7. exp cryotherapy/
8. exp uterine cervix conization/
9. papillomavirus infection/
10. colposcopy/
11. #6 OR #7 OR #8 OR #9 OR #10
12. Cameroon/
13. #5 AND #11 AND #12

**Cochrane library search strategy**

1. [mh^ "Uterine cervical neoplasms”]
2. [mh^ “Cervical intraepithelial neoplasia”]
3. [mh “Uterine Cervical Dysplasia”]
4. ("Cancer du col de l’utérus") : ti, ab, kw
5. ("Cervical carcinoma*") : ti, ab, kw
6. #1 OR #2 OR #3 OR #4 OR #5
7. [mh "Early detection of cancer"]
8. [mh "Cryotherapy"]
9. [mh "Conization"]
10. [mh "Papanicolaou Test"]
11. [mh^ "Colposcopy"]
12. [mh "Human Papillomavirus DNA Test"]
13. (screen-and-treat): ti, ab, kw
14. (prevent*): ti, ab, kw
15. (dépistage): ti, ab, kw
16. ("thermal ablation"): ti, ab, kw
17. (LEEP): ti, ab, kw
18. (LLETZ): ti, ab, kw
19. ("Cold knife"): ti, ab, kw
20. #7 OR #8 OR #9 OR #10 OR #11 OR #13 OR #14 OR #15 OR #16 OR #17 OR #18 OR #19
21. [mh^ "Cameroon"]
22. (Cameroun): ti, ab, kw
23. #21 OR #22
24. #6 AND #20 AND #23

**CINAHL search strategy**

1. (MH “Cervical Intraepithelial Neoplasia+”) OR (MH “Cervix Neoplasms”)
2. (MH “Cervix Dysplasia”)
3. (TI "cancer du col de l’utérus" OR AB "cancer du col de l’utérus")
4. (TI "cervical carcinoma*" OR AB “cervical carcinoma*”)
5. #1 OR #2 OR #3 OR #4
6. (MH “Early detection of cancer”)
7. (MH “Cryotherapy”)
8. (MH “Conization”)
9. (MH “Colposcopy”)
10. (TI “Papanicolaou test*” OR AB “Papanicolaou test*”)
11. (TI “Human Papillomavirus DNA test*” OR AB “Human Papillomavirus DNA test*”)
12. (TI “screen-and-treat” OR AB “screen-and-treat”)
13. (TI “prevent*” OR AB “prevent*”)
14. (TI “dépistage” OR AB “dépistage")
15. (TI "thermal ablation" OR AB “thermal ablation”)
16. (TI “LEEP” OR AB “LEEP”)
17. (TI “LLETZ” OR AB “LLETZ”)
18. (TI “Cold Knife” OR AB “Cold Knife”)
19. #6 OR #7 OR #8 OR #9 OR #10 OR #11 OR #12 OR #13 OR #14 OR #15 OR #16 OR #17 OR #18
20. (MH “Cameroon”)
21. (TI “Cameroun” OR AB “Cameroun”)
22. #20 OR #21
23. #5 AND #19 AND #22

**Web of science search strategy**

We selected the “Topic” field for each row.

1. “cervical intraepithelial neoplasia” OR “uterine cervical dysplasia” OR “cervical neoplasm*” OR “cervical cancer*” OR “cancer du col de l’utérus” OR “cervical carcinoma*” OR “cervical tumo*”
2. “early detection of cancer” OR cryotherapy OR conization OR “Papanicolaou test” OR “colposcopy” OR “Human Papillomavirus DNA test” OR “screen-and-treat” OR “cancer screen*” OR “thermal ablation” OR preventi* OR dépistage OR LEEP OR LLETZ OR “cold knife”
3. Cameroon OR Cameroun
4. #1 AND #2 AND #3

**Google scholar search strategy**

We searched “Title words” using the Publish or Perish software (2012 – 2022, the first 100 results)

Cameroon|Cameroun "cervical intraepithelial neoplasia"|"cervical dysplasia"|"cervical cancer"|"cancer du col de l'utérus"| "cancer screening"|cryotherapy|conization|"screen-and-treat"|dépistage|LEEP|"HPV DNA Test"|colposcopy
