# Supplementary material for: Disparities in cervical cancer screening programs in Cameroon: a scoping review of facilitators and barriers to implementation and uptake of screening
Source: Int J Equity Health. 2023 Aug 17;22:156. doi: 10.1186/s12939-023-01942-2 (PMC10433640; doi:10.1186/s12939-023-01942-2)
Supplement: Supplementary file 2 — Additional file 2. [file 12939_2023_1942_MOESM2_ESM.docx]

**APPENDIX 2: Title and abstract screening form**

**Table VII**: Title and abstract screening form

| **Author** | **Year of publication** | **Title** | **Journal details** | **Abstract** | **DOI** | **Name of database** | **Reviewers 1** | | **Reviewer 2** | |
| --- | --- | --- | --- | --- | --- | --- | --- | --- | --- | --- |
|  |  |  |  |  |  |  | **Decision** | **Reasons for exclusion** | **Decision** | **Reasons for exclusion** |
|  |  |  |  |  |  |  |  |  |  |  |
|  |  |  |  |  |  |  |  |  |  |  |
|  |  |  |  |  |  |  |  |  |  |  |
|  |  |  |  |  |  |  |  |  |  |  |
|  |  |  |  |  |  |  |  |  |  |  |
|  |  |  |  |  |  |  |  |  |  |  |
|  |  |  |  |  |  |  |  |  |  |  |
|  |  |  |  |  |  |  |  |  |  |  |
