# Supplementary material for: Disparities in cervical cancer screening programs in Cameroon: a scoping review of facilitators and barriers to implementation and uptake of screening
Source: Int J Equity Health. 2023 Aug 17;22:156. doi: 10.1186/s12939-023-01942-2 (PMC10433640; doi:10.1186/s12939-023-01942-2)
Supplement: Supplementary file 3 — Additional file 3. [file 12939_2023_1942_MOESM3_ESM.docx]

**APPENDIX 3: Data extraction forms**

**Table VIII:** Data extraction form I_Characteristics of included studies

| **Scoping review title:** | | | | | | | | | | | | | |
| --- | --- | --- | --- | --- | --- | --- | --- | --- | --- | --- | --- | --- | --- |
| **Main objective:** | | | | | | | | | | | | | |
| **N°** | **Study site** | | | **Year of publication** | **Author** | **Title** | **Aim** | **Study design** | **Type & N° of participants** | **Data collection methods** | **Study outcomes** | **Screening uptake** | **Post-screening treatment** |
|  | **Region** | **District** | **Setting** |  |  |  |  |  |  |  |  |  |  |
|  |  |  |  |  |  |  |  |  |  |  |  |  |  |
|  |  |  |  |  |  |  |  |  |  |  |  |  |  |
|  |  |  |  |  |  |  |  |  |  |  |  |  |  |
|  |  |  |  |  |  |  |  |  |  |  |  |  |  |
|  |  |  |  |  |  |  |  |  |  |  |  |  |  |
|  |  |  |  |  |  |  |  |  |  |  |  |  |  |
|  |  |  |  |  |  |  |  |  |  |  |  |  |  |
|  |  |  |  |  |  |  |  |  |  |  |  |  |  |
|  |  |  |  |  |  |  |  |  |  |  |  |  |  |
|  |  |  |  |  |  |  |  |  |  |  |  |  |  |

**Table IX**: Data collection form 2_Facilitators and barriers to the implementation of cervical cancer screening programs

| **Item** | **Barriers** | **Studies reporting barriers** | **Facilitators** | **Studies reporting facilitators** |
| --- | --- | --- | --- | --- |
| **Screening test** |  |  |  |  |
| General screening for cervical cancer |  |  |  |  |
| VIA/VILI |  |  |  |  |
| Cervical HPV test |  |  |  |  |
| Cytology/Pap smear |  |  |  |  |
|  |  |  |  |  |
| **Treatment for precancerous lesions** |  |  |  |  |
| General screening methods |  |  |  |  |
| Cryotherapy |  |  |  |  |
| Thermocoagulation |  |  |  |  |
| Loop Electrosurgical Excision Procedure (LEEP) |  |  |  |  |
|  |  |  |  |  |
